# Simultaneous Gemcitabine and Percutaneous CT-Guided Irreversible Electroporation for Locally Advanced Pancreatic Cancer

**DOI:** 10.1155/2022/3523769

**Published:** 2022-06-14

**Authors:** Yangyang Ma, Yanli Xing, Hongmei Li, Bing Liang, Rongrong Li, Jianyu Li, Zhonghai Li, Mao Lin, Lizhi Niu

**Affiliations:** ^1^Central Laboratory, Fuda Cancer Hospital, Guangzhou 510665, China; ^2^Department of Oncology, Fuda Cancer Hospital, Guangzhou 510665, China; ^3^Department of Surgery and Anesthesia, Fuda Cancer Hospital, Guangzhou 510665, China; ^4^Department of Ultrasound, Fuda Cancer Hospital, Guangzhou 510665, China; ^5^Department of Radiology, Fuda Cancer Hospital, Guangzhou 510665, China

## Abstract

**Background:**

Irreversible electroporation (IRE) is a new local ablation technique for pancreatic cancer. The aim of this study is to analyse the safety and effectiveness of simultaneous gemcitabine and percutaneous CT-guided IRE for locally advanced pancreatic cancer (LAPC).

**Materials and Methods:**

From October 2016 to January 2018, 61 patients with LAPC who received simultaneous gemcitabine and IRE therapy (GEM-IRE group, *n* = 31) or IRE alone therapy (IRE group, *n* = 30). Routine intravenous gemcitabine chemotherapy was performed 2 weeks after IRE in both groups.

**Results:**

Technical success rates were 90.0% (27/30) and 93.3% (28/30) in the GEM-IRE and IRE groups. Compared with the IRE group, the GEM-IRE group exhibited longer overall survival (OS), local tumor progression free survival (LTPFS), and distant disease free survival (DDFS) from IRE (OS, 17.1 *vs.* 14.2 months, *p*=0.031; LTPFS, 14.6 *vs.* 10.2 months, *p*=0.045; DDFS, 15.4 *vs.* 11.7 months, *p*=0.071). Multivariate Cox regression analysis results suggested that tumor volume ≤37 cm^3^ and simultaneous gemcitabine with IRE were significant independent prognostic factors of OS, LTPFS, and DDFS. Four major adverse reactions occurred; all of them were resolved after symptomatic treatment.

**Conclusions:**

Simultaneous gemcitabine and percutaneous CT-guided IRE therapy model was effective and well-tolerated therapeutic strategy in LAPC patients.

## 1. Introduction

It is projected that pancreatic cancer will become the second leading cause of death in the United States and Germany within the next decade [1, 2]. Approximately 80% of patients are diagnosed with locally advanced pancreatic cancer (LAPC), and in fewer than 20% of these patients, surgical resection is possible [3, 4], and a 5-year survival rate is less than 8% [5]. First-line chemotherapy regimens for LAPC include FOLFIRINOX, a combination of gemcitabine and albumin-bound paclitaxel, or just gemcitabine. However, the LAPC prognosis remains dismal, even with chemotherapy.

At present, minimally invasive treatments such as radiofrequency, microwave, and cryoablation have been demonstrated to be effective for LAPC patients [6–8]. These types of technology, however, have the potential to damage bile ducts, pancreatic ducts, peripancreatic vessels, and the duodenum, which may result in limited benefit [9, 10]. Irreversible electroporation (IRE) is a novel, minimally invasive ablation technique for soft tissue cancer, which uses high-voltage electrical pulses to develop irreversible small holes on the surface of the cell membrane, causing a disruption of homeostasis and cell death [11–14]. Currently, IRE has demonstrated distinct superiority and potency in the management of LAPC [15–18].

In recent years, there has been an explosion in the use of electrochemical therapy (ECT) in the treatment of solid tumors [19–21]. Usually, ECT combines poorly or not penetrating but strongly available cytotoxic agents with electroporation. Because it strengthens the transmission of molecules to the structure, ECT has a high potential for treating advanced solid tumors [22, 23]. In the reversible electroporation (RE) zone, cell membrane permeability due to electroporation can promote drug diffusion into the cells [24–27], which may improve antitumor efficacy. On the other hand, a preclinical study has shown that IRE enhanced gemcitabine delivery into the RE zone and thereby reduced local recurrence [28].

Based on the above studies, a retrospective study about simultaneous gemcitabine and percutaneous CT-guided IRE for LAPC was conducted. In this study, we evaluated overall survival (OS), local tumor progression free survival (LTPFS), distant disease free survival (DDFS), and objective response rate (ORR) and adverse events after combination therapy, towards a more effective treatment strategy for LAPC.

## 2. Materials and Methods

### 2.1. Patients

The patients received either simultaneous gemcitabine and percutaneous CT-guided IRE (GEM-IRE group) or IRE alone (IRE group). We executed this retrospective study with the approval of the Ethics Committee of Fuda Cancer Hospital and strict administration of the Declaration of Helsinki and the Declaration of Good Clinical Practice. We obtained written informed consent from each patient.

### 2.2. Inclusion and Exclusion Criteria

Inclusion Criteria: (1) patients older than 18 years; (2) expected survival >3 months; (3) tumor diameter ≤5 cm; (4) sufficient hepatic, renal, and bone marrow function were maintained; (5) performance status score of ≤2; (6) patients could not undergo surgical resection, or patients who could undergo surgery but prefer IRE treatment; (7) informed consent. Exclusion Criteria: (1) severe cardiac, pulmonary, and renal insufficiency or inability to tolerate general anesthesia with endotracheal intubation; (2) allergy to contrast media; (3) chemotherapy or radiotherapy within 1 month prior to the procedure; (4) history of epilepsy; and (5) history of heart arrhythmia, implantation of metallic stent or cardiac pacemaker.

### 2.3. Procedure

In all patients, preoperative bowel preparation was performed and anesthesia was inducted with etomidate (0.3 mg/kg), remifentanil (3–5 *μ*g/kg, 1.2–1.6 mg/h), and cisatracurium besilate (0.1 mg/kg). Anesthesia was administered by injection of intravenous cisatracurium besilate, remifentanil, and propofol with sevoflurane inhalation. To avoid cardiac arrhythmias, an electrocardiogram (ECG)-gating device was synchronized with pulse delivery during the cardiac refractory period. To prevent generalized muscle contractions, complete muscle relaxants were administered immediately before IRE delivery.

In the GEM-IRE group, before IRE treatment, gemcitabine (1000 mg/m^2^) was given intravenously for 30 minutes. Once-weekly infusions of gemcitabine were administered for the first two weeks, followed by a one-week rest period. Subsequent cycles consisted of weekly injections for three continuous weeks, with a cycle of four weeks each. The therapy was maintained until disease progression, by mRECIST or intolerable toxicity [29]. Upon disease progression, palliative care was given.

During IRE, CT scanning and ultrasound were used to guide percutaneous insertion of the electrode. Pretreatment planning determines electrode-insertion mode, electrode number, and intraoperative parameters. The IRE parameters were set up as follows: 90-microsecond high-voltage (1500–3000 V), delivered between 9 pairs of paired monopolar electrodes with 2 cm exposed tips, totaling 90 pulses. Further treatment includes anti-infection treatments, stomach-preserving and liver protection therapies, and nutritional treatment.

### 2.4. Carbohydrate Antigen 19-9 (CA19-9)

Carbohydrate antigen 19-9 (CA19-9) was analyzed 30 days postintervention to evaluate the initial effect.

### 2.5. Technical Success

Technical success was considered to be the ability to successfully deliver pulses (at least 90) to ensure a change in current of at least 5 A relative to the initial 10 pulses.

### 2.6. Follow-Up and Response Assessment

Imaging follow-up was done using contrast-enhanced CT or magnetic resonance imaging (MRI) at 1, 3, and 6 months postintervention and then every 3 months. Imaging follow-up at 1 month evaluated the success of the technique. Imaging efficacy evaluation was based on mRECIST [29]. Complete response (CR) was considered as the absence of arterial enhancing lesions. A partial response (PR) was indicated by a 30% decrease in the size of the target lesion. Progressive disease (PD) was considered by a decrease of 20% in the size of the lesion. Stable disease (SD) was considered by lack of target lesions reduction to PR or increase to PD. We estimated the objective response rate (ORR) as ORR = (CR + PR)/total number × 100%.

### 2.7. OS

The OS was the length of time from diagnosis and IRE treatment to death from any cause. The LTPFS was considered as the time from IRE treatment to local tumor progression. DDFS was considered as the time from IRE treatment to distant metastasis.

### 2.8. Safety

Adverse events were assessed after 24 h using enhanced CT and ultrasound. Any adverse events that occurred within 30 days of treatment were graded depending on the CTCAE V4.0. CTCAE grades III-IV were classified as “severe.” The change of AST, ALT, and ALP and routine blood tests were taken before the procedure and were repeated 1, 3, 7, and 14 days after the procedure to evaluate intervention safety.

### 2.9. Statistical Analysis

Statistical analyses were performed with GraphPad Prism and SPSS 25.0 (IBM). We used the Fisher's exact test, the Mann–Whitney test, and the log-rank test to compare continuous data, categorical data, and survival curves. Wilcoxon matching was used to compare consecutive data tests in the same group. All statistical tests were two-sided. A *P* value of 0.05 indicates statistical significance.

## 3. Results

### 3.1. Patient Parameters

**Figure 1:**
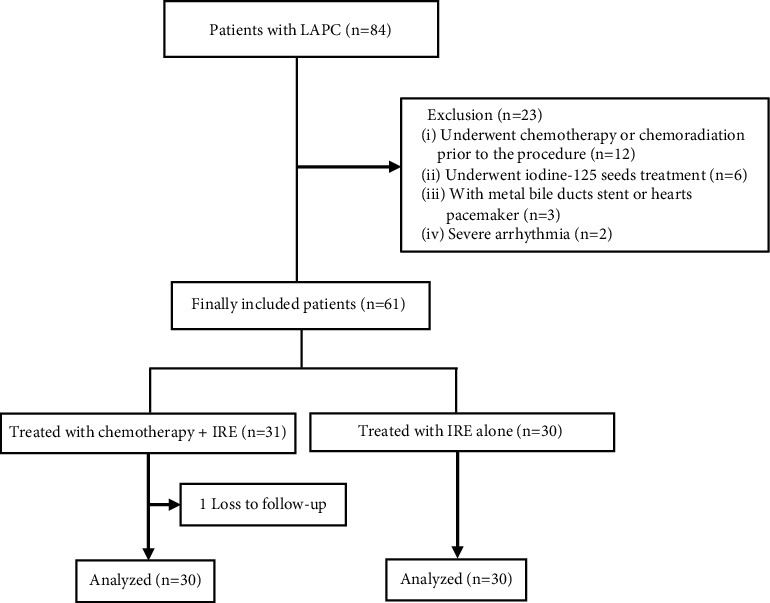
Flow diagram of patients included in the study.

From October 2016 to January 2018, 84 patients were included in our study (Figure 1). Among them, 23 patients were excluded because of chemoradiation within 1 month prior to the procedure (*n* = 12), iodine-125 seed treatment (*n* = 6), with metal duct stent or heart pacemaker (*n* = 3), and severe arrhythmia (*n* = 2). Overall, the GEM-IRE group (31 patients) received gemcitabine and IRE simultaneously, and the IRE group (30 patients) received IRE alone. During the follow-up, 1 patient lost to follow in the GEM-IRE group. Finally, 60 patients were analyzed. The clinical features of the 60 included patients are given in Table 1.

### 3.2. Tumor Markers by CA19-9

**Figure 2:**
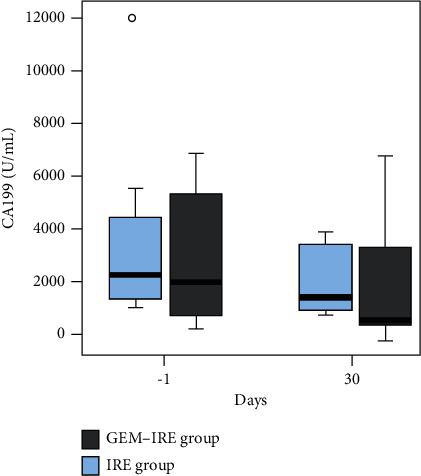
CA19-9 levels before and after treatment.

The positive serum CA19-9 level rates in the GEM-IRE and IRE groups were 73.3% (22/30) and 80.0% (24/30), respectively. The CA19-9 levels in the GEM-IRE and IRE groups before treatment had significantly decreased by 1 month posttreatment (1981.5 *vs.* 752.5, *p*=0.005, and 2376 *vs*. 1554, *p*=0.012, respectively); no statistically significant difference in both groups before treatment or 1 month after treatment (*p*=0.567 and *p*=0.053, respectively; Figure 2).

### 3.3. Treatment Success Rate and Tumor Response

**Figure 3:**
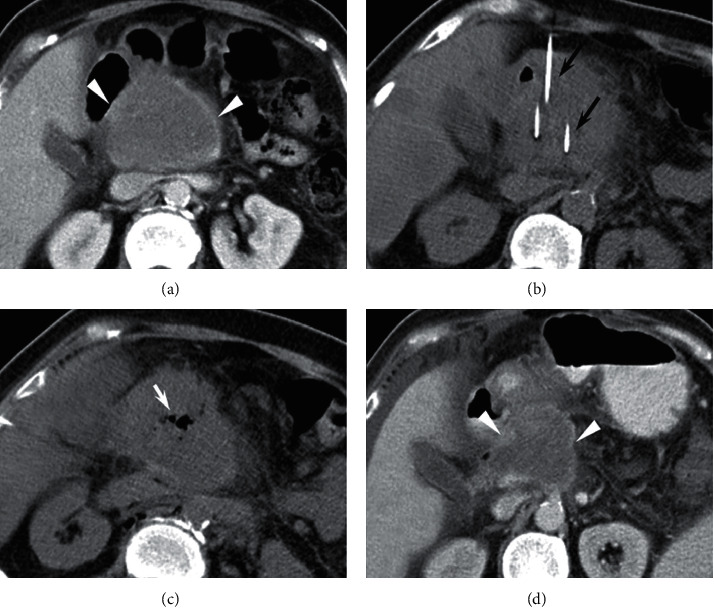
A 66-year-old female with unresectable pancreatic head tumors treated with gemcitabine and irreversible electroporation (GEM-IRE) simultaneously. (a) Preoperative contrast-enhanced CT showed tumor size (7.4 × 6.2 cm). (b) Arrows indicate electrode probes. (c) Bubbles were seen immediately after IRE. (d) 3 months after operation, preoperative contrast-enhanced CT revealed a tumor size of 4.6 × 5.1 cm.

There was no operative mortality within 90 days. Technical success rates were 90.0% (27/30) in the GEM-IRE group and 93.3% in the IRE group (28/30), (*p* > 0.05). The 1-, 3-, and 6-month ORRs for the GEM-IRE group *vs*. the IRE group were 93.3%, 83.3%, and 65.5% *vs*. 86.6%, 70.0%, and 53.8%, respectively. A representative CT result from a GEM-IRE patient is displayed in Figure 3.

### 3.4. OS

**Figure 4:**
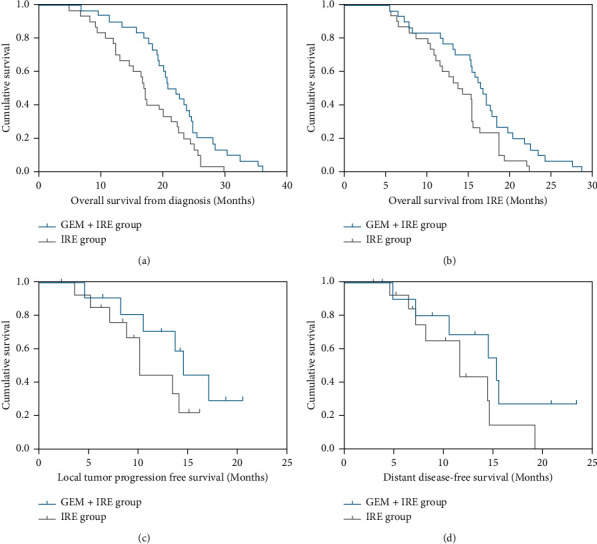
Kaplan–Meier survival curves. (a) The median OS from diagnosis. (b) The median OS from IRE. (c) Local tumor progression free survival from IRE. (d) Distant disease-free survival from IRE.

The median follow-up time was 20.4 months (3.0–28.4 months). The median OS from diagnosis in the GEM-IRE group was significantly longer than that of those in the IRE group (21.5 *vs.* 16.7 months, respectively; hazard ratio (HR), 0.520; *p*=0.019; Figure 4(a)). The median OS from IRE was also significantly longer in the GEM-IRE group than that of those in the IRE group (17.1 *vs.* 14.2 months, respectively; HR, 0.54; *p*=0.031; Figure 4(b)). According to univariate analysis, tumor size (HR = 1.925, 95% CI, 1.056–3.559, *p*=0.046), tumor volume (HR = 2.486, 95% CI, 1.245–4.325, *p*=0.009), local recurrence (HR = 3.522, 95% CI, 1.362–7.023, *p*=0.006), and IRE treatment (HR = 0.389, 95% CI, 0.178–0.952, *p*=0.045) were related to OS. In addition, the independent prognostic factors were tumor volume (HR = 2.675, 95% CI, 1.230–7.215, *p*=0.035) and IRE treatment (HR = 0.422, 95% CI, 0.157–0.958, *p*=0.007) (Table 2).

### 3.5. LTPFS

A total of 25 patients experienced tumor progression, including 11 (36.7%) patients in the GEM-IRE group and 14 (46.6%) patients in the IRE group (*p*=0.436). The median LTPFS for patients in GEM-IRE and IRE groups were 14.6 months and 10.2 months, respectively (*p*=0.045, Figure 4(c)). Univariate analysis for LTPFS revealed that tumor volume (HR = 2.356, 95% CI, 1.367–3.161, *p*=0.001), local recurrence (HR = 0.445, 95% CI, 0.245–0.975, *p*=0.024), CA19-9 level (HR = 2.156, 95% CI, 1.034–4.265, *p*=0.045), and IRE treatment (HR = 0.545, 95% CI, 0.326–1.051, *p*=0.047) were associated with LTPFS. In addition, the independent prognostic factors were tumor volume (HR = 1.683, 95% CI, 1.035–3.191, *p*=0.032) and IRE treatment (HR = 0.556, 95% CI, 0.315–1.260, *p*=0.043) (Table 3).

### 3.6. DDFS

The median DDFS were 15.4 months and 11.7 months in the GEM-IRE and IRE groups, respectively (*p*=0.071, Figure 4(d)). According to the univariate and multivariate analyses, tumor volume (HR = 2.364, 95% CI, 1.059–4.685, *p*=0.007), local recurrence (HR = 3.432, 95% CI, 1.406–8.125, *p*=0.006), and IER treatment (HR = 0.326, 95% CI, 0.192–0.752, *p*=0.014) were associated with DDFS. Moreover, tumor volume (HR = 2.856, 95% CI, 1.196–7.398, *p*=0.024) and IRE treatment (HR = 0.385, 95% CI, 0.202–0.654, *p*=0.021) were found to be an independent favourable factor of DDFS (Table 4).

### 3.7. Safety

Within 30 days post-IRE, there were 16 minor adverse reactions and 4 major adverse reactions (Tables 5 and 6). The major adverse reactions included two cases of pancreatitis (grade III), one case of severe neutropenia (grade IV), and one case of gastroduodenal artery hemorrhage (grade IV) in the GEM-IRE group. The case of severe neutropenia was diagnosed on day 5 and that of gastroduodenal artery hemorrhage on day 11 after IRE. After a subcutaneous injection of 300 *μg* of recombinant human granulocyte colony-stimulating factor (G-CSF), the neutrophil level increased to 5.6 × 10^9^/L within 24 hours. The gastroduodenal artery hemorrhage cases were controlled by vascular interventional embolization with 560–710 *μ*m of gelatin sponge particles and 2 mm and 3 mm coils. The differences in the frequencies of adverse events were not statistically significant in both groups (*p* > 0.05 for all; Table 6). The 90-day mortality rate was 0% overall.

**Figure 5:**
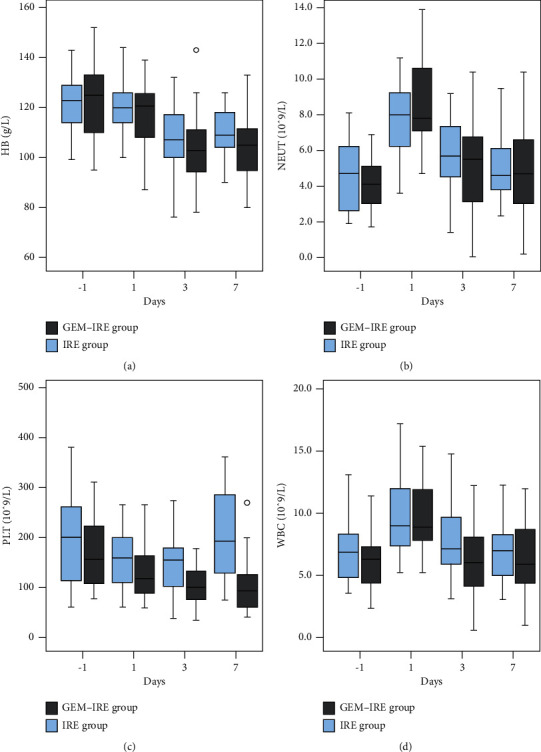
Changes in routine blood tests before and after treatment in both groups. (a) Changes in hemoglobin levels. (b) Changes of neutrophil counts. (c) Changes in platelet count. (d) Changes in white blood cell count.

For routine blood tests, the postoperative hemoglobin level continued to significantly decrease in both groups on days 3 and 7 (*p* < 0.000, Figure 5(a)). The neutrophil counts showed a significant increase on day 1 (*p* < 0.000, Figure 5(b)); however, the levels recovered to preoperative levels by day 3, 7. There were significant differences in the platelet counts (*p*=0.0035, Figure 5(c)) and white blood cell counts (*p*=0.0179, Figure 5(d)) after treatment between the two groups at days 3 and 7 (*p*=0.0035) (Figure 5).

**Figure 6:**
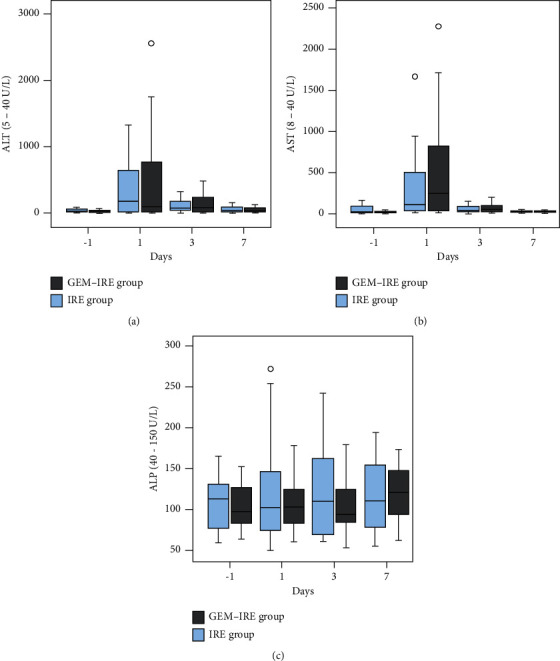
Changes in liver function before and after treatment. (a) ALT levels. (b) AST levels. (c) Changes in ALP levels.

For liver function, on postoperation day 1, ALT (Figure 6(a)) and AST (Figure 6(b)) values for both groups indicated a significant rising (*p* < 0.000 for both), but the levels decreased rapidly on day 3 and recovered to preoperative levels by day 7. Interestingly, the two groups did not differ statistically significantly (*p* > 0.05 for all). No obvious changes in alkaline phosphatase (ALP) (Figure 6(c)) were observed after treatment.

## 4. Discussion

Recent chemotherapy regimens have improved OS for LAPC patients only by single-digit months [30]. Due to the insensitivity to conventional chemotherapy, gemcitabine administration is suppressed by the peritumor stroma and pancreatic cancer patients with low pancreatic hENT1 levels exhibit a significantly lower response to gemcitabine. To overcome this obstacle, ECT was developed. ECT utilizes high-intensity electrical pulses to enhance cell membrane permeability to injected chemotherapies. Although ECT has been applied to solid tumors for the past 2 decades and has been proven effective [31, 32], this was the first study to investigate simultaneous gemcitabine combination with IRE. Recent studies suggest that IRE combination with chemotherapy improves survival, but they were all induction chemotherapy regimens before IRE or postoperative adjuvant chemotherapy [33–35].

Our study was the first to demonstrate the antitumor effects of simultaneously combining gemcitabine with IRE. Our data showed that the GEM-IRE group had a longer OS (21.5 months). According to statistics, the median OS ranged from 17.9–27.0 months in some previous retrospective studies [34, 36–38]. In the aforementioned retrospective studies, patients who underwent induction chemotherapy achieved stable disease. Thus, simultaneous gemcitabine and IRE treatment had some survival benefit regardless of patient condition. Using percutaneous IRE as first-line treatment for LAPC without prior systemic treatment, Mansson et al. [39] found that the median OS after diagnosis was 13.3 months compared to 21.5 months in our study, indicating that simultaneous gemcitabine and IRE achieved better survival than percutaneous IRE as first-line LAPC treatment.

Interestingly, although the treatment success rate was similar within both groups, the short-term tumor response in the GEM-IRE group was higher than in the IRE group. Therefore, this study showed that the simultaneous treatment of gemcitabine and IRE is a better option for patients with LAPC. The main reason for this phenomenon might be that the peak plasma values of gemcitabine and its deaminated metabolite occurr within 30 minutes of injection. Continued treatment with IRE at this point prolonged its peak value and enhanced drug toxicity. This is consistent with findings by Shamseddine et al. that intra-arterial plasma gemcitabine peaks at 30 minutes [40]. Membrane permeation by electroporation enhances drug entry into the cell, enhancing cytotoxicity [26, 27, 41, 42]. These factors highlight the potential value of ECT in LAPC treatment.

IRE enhances drug transmission to cells via breaking up the dense mesenchymal tissues of tumors [28, 43]. Considering LAPC's high heterogeneity, local control of the tumor by RE ablation followed by neoadjuvant chemotherapy may improve survival rates. Thus, neoadjuvant chemotherapy followed by simultaneous gemcitabine and IRE may benefit LAPC patients more.

In terms of safety, most of the postoperative adverse reactions seen in this study were classified as grade I or II based on CTCAE V4.0 standards, while 4 cases were considered severe (grade III or grade IV). Between the 2 groups, there were no significant differences of adverse reactions (*p* > 0.05). Although IRE is known to preserve the structure of blood vessels, we observed a case of hemorrhage in the gastroduodenal artery during follow-up. Similar complications have been reported in other studies of about 4–7% [33–35, 44]. This may be because the duodenum was infiltrated by a pancreatic tumor and IRE ablation caused a duodenal ulcer, allowing gastric acid exudation and, ultimately, blood vessel rupture and bleeding. Thus, the combined use of laparotomy and gastrointestinal anastomosis may be an effective preventive method. Additionally, after the first postintervention day, ALT and AST levels increased significantly, suggesting that neither treatment has long-term effects on liver function and that the combination therapy was safe for LAPC treatment.

The findings of our study have a few limitations. First is small sample size. In the future, multicenter, prospective, randomized controlled, large-sample clinical studies are needed to support our results. Second, our study did not directly compare our treatments with conventional chemotherapy, including FOLFIRINOX, gemcitabine-based chemotherapy. Thus, larger, multicenter studies are being developed to support our results.

## Figures and Tables

**Table 1:** Patients characteristics (*n* (%)).

Characteristic	GEM-IRE group (*n* = 30)	IRE group (*n* = 30)	*P* value
Sex			
F	16 (53.3)	18 (60.0)	0.795
M	14 (46.7)	12 (40)	

Mean age (years)	61 (43–78)	67 (47–75)	
Lesion size (cm)	3.8 (2.1–4.7)	4.1 (2.3–4.6)	
Tumor location			0.763
Head	7 (23.3)	8 (26.7)	
Body	12 (40.0)	15 (50.0)	
Tail	7(23.3)	4 (13.3)	
Uncinate process	4 (13.4)	3 (10.0)	

Preoperative surgical therapy			0.624
None	23 (76.6)	25 (83.4)	
Gastrojejunostomy	2 (6.7)	1 (3.3)	
Hepaticojejunostomy	1 (3.3)	1 (3.3)	
Double bypass	2 (6.7)	0 (0.0)	
Others	2 (6.7)	3 (10.0)	

PS			0.627
0	8 (26.7)	5 (16.7)	
1	18 (60.0)	22 (73.3)	
2	4 (13.3)	3 (10.0)	

PS: performance status.

**Table 2:** Univariate and multivariate analyses of overall survival.

Characteristic		Univariate analysis		Multivariate analysis	
		HR (95% CI)	*P* value	HR (95% CI)	*P* value
Age	≤60/>60	1.245 (0.625–2.856)	0.156		
Sex	Female/male	0.653 (0.302–1.127)	0.129		
Tumor site	Head/body/tail	0.936 (0.648–1.582)	0.832		
Tumor size (cm)	≤5/>5	1.925 (1.056, 3.559)	0.046	1.369 (0.625, 2.848)	0.521
Tumor volume (cm^3^)	≤37/>37	2.486 (1.245–4.325)	0.009	2.675 (1.230–7.215)	0.035
Local recurrence	≤6 mo/>6 mo	3.522 (1.362–7.023)	0.006	0.542 (0.308–2.034)	0.384
WBC (×10^9^)	≤10/>10	1.352 (0.475–2.322)	0.789		
HGB (g/L)	≤120/>120	1.086 (0.365–1.458)	0.426		
PLT (× 10^9^)	≤300/>300	1.335 (0.468–3.589)	0.459		
ALT (U/L)	≤40/>40	0.895 (0.257–1.752)	0.658		
AST (U/L)	≤40/>40	0.235 (0.128–1.329)	0.457		
ALP (U/L)	≤100/>100	0.965 (0.042–1.436)	0.689		
CA19-9 level	≤35/>35	1.350 (0.618–3.572)	0.245		
Complications	Yes/no	0.621 (0.321–1.126)	0.096		
IRE	With/without GEM	0.389 (0.178–0.952)	0.045	0.422 (0.157–0.958)	0.007

IRE: irreversible electroporation; GEM: gemcitabine; HR: hazard ratio; WBC: white blood cell count; HGB: hemoglobin; PLT: platelet count; ALT: alanine transaminase; AST: aspartate aminotransferase; ALP: alkaline phosphatase; CA19-9: carbohydrate antigen 19-9.

**Table 3:** Univariate and multivariate analyses of local tumor progression free survival.

Characteristic		Univariate analysis		Multivariate analysis	
		HR (95% CI)	*P* value	HR (95% CI)	*P* value
Age	≤60/>60	0.654 (0.565–1.818)	0.185		
Sex	Female/male	1.588 (0.927–2.579)	0.056		
Tumor site	Head/body/tail	1.167 (0.6987–1.640)	0.960		
Tumor size (cm)	≤5/>5	1.154 (0.776–1.705)	0.512		
Tumor volume (cm^3^)	≤37/>37	2.356 (1.367–3.161)	0.001	1.683 (1.035–3.191)	0.032
Local recurrence	≤6 mo/>6 mo	0.445 (0.245–0.975)	0.024	0.526 (0.225–1.325)	0.151
WBC (×10^9^)	≤10/>10	1.154 (0.479–2.658)	0.811		
HGB (g/L)	≤120/>120	0.673 (0.318–1.535)	0.369		
PLT (×10^9^)	≤300/>300	0.623 (0.256–1.338)	0.206		
ALT (U/L)	≤40/>40	0.741 (0.325–1.437)	0.356		
AST (U/L)	≤40/>40	0.678 (0.328–1.478)	0.246		
ALP (U/L)	≤100/>100	0.823 (0.446–1.489)	0.744		
CA19-9 level	≤35/>35	2.156 (1.034–4.265)	0.045	1.916 (0.931–3.568)	0.078
Complications	Yes/no	0.645 (0.334–1.451)	0.095		
IRE	With/without GEM	0.545 (0.326–1.051)	0.047	0.556 (0.315–1.260)	0.043

IRE: irreversible electroporation; GEM: gemcitabine; HR: hazard ratio; WBC: white blood cell count; HGB: hemoglobin; PLT: platelet count; ALT: alanine transaminase; AST: aspartate aminotransferase; ALP: alkaline phosphatase; CA19-9: carbohydrate antigen 19-9.

**Table 4:** Univariate and multivariate analyses of distance disease-free survival.

Characteristic		Univariate analysis		Multivariate analysis	
		HR (95% CI)	*P* value	HR (95% CI)	*P* value
Age	≤60/>60	1.214 (0.985–1.125)	0.421		
Sex	Female/male	0.621 (0.34–1.263)	0.127		
Tumor site	Head/body/tail	0.772 (0.438–1.248)	0.337		
Tumor size (cm)		1.187 (0.674–2.062	0.543		
Tumor volume (cm^3^)	≤37/>37	2.364 (1.059–4.685)	0.007	2.856 (1.196–7.398)	0.024
Local recurrence	≤6 mo/>6 mo	3.432 (1.406–8.125)	0.006	0.523 (0.135–1.420)	0.231
WBC (×10^9^)	≤10/>10	1.128 (0.364–2.825)	0.858		
HGB (g/L)	≤120/>120	0.644 (0.345–1.670)	0.403		
PLT (×10^9^)	≤300/>300	1.218 (0.578–2.567)	0.628		
ALT (U/L)	≤40/>40	0.767 (0.358–1.716)	0.504		
AST (U/L)	≤40/>40	0.484 (0.205–1.561)	0.176		
ALP (U/L)	≤100/>100	0.921 (0.546–1.950)	0.703		
CA19-9 level	≤35/>35	1.638 (0.720–3.882)	0.225		
Complications	Yes/no	1.382 (0.678–2.821)	0.363		
IRE	With/without GEM	0.326 (0.192–0.752)	0.014	0.385 (0.202–0.654)	0.021

IRE: irreversible electroporation; GEM: gemcitabine; HR: hazard ratio; WBC: white blood cell count; HGB: hemoglobin; PLT: platelet count; ALT: alanine transaminase; AST: aspartate aminotransferase; ALP: alkaline phosphatase; CA19-9: carbohydrate antigen 19-9.

**Table 5:** The type and treatment of adverse reactions (*n* = 60).

Adverse reactions	Grade I/II	Grade III	Grade IV	Treatment
Hemoglobin reduction	37	0	0	NA
Leukocyte reduction	6	0	0	NA
Neutropenia	4	0	1	Human granulocyte colony-stimulating factor
Thrombocytopenia	27	0	0	NA
Transient elevation of myocardial enzyme	22	0	0	NA
Proteinuria	1	0	0	NA
Hypokalemia	3	0	0	NA
Pancreatitis	0	2	0	Drainage and antibiotics
Bleeding from duodenal ulcer	0	0	1	Interventional embolization
Fever	10	0	0	Antibiotics
Diarrhea	1	0	0	Pancreatic enzyme suppletion
Nausea and vomiting	9	0	0	Antiemetics
Infection	3	0	0	Antibiotics and drainage
Abdominal pain	39	0	0	Oral analgesics
Loss of appetite and	9	0	0	Nasojejunal tube feeding
Mild ascites	27	0	0	NA
Mild pleural effusion	8	0	0	NA
Abdominal distention	12	0	0	NA

NA: no receiving further treatment.

**Table 6:** Adverse reaction rates after treatment (*n* (%)).

Characteristic	GEM-IRE group (*n* = 30)	IRE group (*n* = 30)	*P* value
Hemoglobin reduction	22 (73.3)	15 (50.0)	0.110
Leukocyte reduction	4 (13.3)	2 (6.7)	0.671
Neutropenia	2 (6.7)	3 (10.0)	1.000
Thrombocytopenia	15 (50.0)	12 (40.0)	0.604
Transient elevation of myocardial enzyme	14 (46.7)	8 (26.7)	0.180
Proteinuria	1 (3.3)	0 (0.0)	1.000
Hypokalemia	1 (3.3)	2 (6.7)	1.000
Pancreatitis	1 (3.3)	1 (3.3)	1.000
Bleeding from duodenal ulcer	1 (3.3)	0 (0.0)	1.000
Fever	6 (20.0)	4 (13.3)	0.731
Diarrhea	1 (3.3)	0 (0.0)	1.000
Nausea and vomiting	6 (20.0)	3 (10.0)	0.472
Infection	2 (6.7)	1 (3.3)	1.000
Abdominal pain	5 (16.7)	7 (23.3)	0.748
Loss of appetite and/or reduced intake	7 (23.3)	2 (6.7)	0.145
Mild ascites	15 (50.0)	12 (40.0)	0.604
Mild pleural effusion	5 (16.7)	3 (10.0)	0.706
Abdominal distention	8 (26.7)	4 (13.3)	0.333

## Data Availability

All data are available upon reasonable request.
